# Outbreak Associated with SARS-CoV-2 B.1.617.2 (Delta) Variant in an Elementary School — Marin County, California, May–June 2021

**DOI:** 10.15585/mmwr.mm7035e2

**Published:** 2021-09-03

**Authors:** Tracy Lam-Hine, Stephen A. McCurdy, Lisa Santora, Lael Duncan, Russell Corbett-Detig, Beatrix Kapusinszky, Matthew Willis

**Affiliations:** ^1^County of Marin Department of Health and Human Services, San Rafael, California; ^2^UC Berkeley School of Public Health, University of California, Berkeley, California; ^3^UC Davis School of Medicine, University of California, Sacramento, California; ^4^Baskin School of Engineering, University of California, Santa Cruz, California; ^5^Napa-Solano-Yolo-Marin-Mendocino County Public Health Laboratory, Fairfield, California.

On May 25, 2021, the Marin County Department of Public Health (MCPH) was notified by an elementary school that on May 23, an unvaccinated teacher had reported receiving a positive test result for SARS-CoV-2, the virus that causes COVID-19. The teacher reported becoming symptomatic on May 19, but continued to work for 2 days before receiving a test on May 21. On occasion during this time, the teacher read aloud unmasked to the class despite school requirements to mask while indoors. Beginning May 23, additional cases of COVID-19 were reported among other staff members, students, parents, and siblings connected to the school. To characterize the outbreak, on May 26, MCPH initiated case investigation and contact tracing that included whole genome sequencing (WGS) of available specimens. A total of 27 cases were identified, including that of the teacher. During May 23–26, among the teacher’s 24 students, 22 students, all ineligible for vaccination because of age, received testing for SARS-CoV-2; 12 received positive test results. The attack rate in the two rows seated closest to the teacher’s desk was 80% (eight of 10) and was 28% (four of 14) in the three back rows (Fisher’s exact test; p = 0.036). During May 24–June 1, six of 18 students in a separate grade at the school, all also too young for vaccination, received positive SARS-CoV-2 test results. Eight additional cases were also identified, all in parents and siblings of students in these two grades. Among these additional cases, three were in persons fully vaccinated in accordance with CDC recommendations (*1*). Among the 27 total cases, 22 (81%) persons reported symptoms; the most frequently reported symptoms were fever (41%), cough (33%), headache (26%), and sore throat (26%). WGS of all 18 available specimens identified the B.1.617.2 (Delta) variant. Vaccines are effective against the Delta variant (*2*), but risk of transmission remains elevated among unvaccinated persons in schools without strict adherence to prevention strategies. In addition to vaccination for eligible persons, strict adherence to nonpharmaceutical prevention strategies, including masking, routine testing, facility ventilation, and staying home when symptomatic, are important to ensure safe in-person learning in schools (*3*).

## Investigation and Findings

The outbreak location was an elementary school in Marin County, California, which serves 205 students in prekindergarten through eighth grade and has 24 staff members. Each grade includes 20 to 25 students in single classrooms. Other than two teachers, one of whom was the index patient, all school staff members were vaccinated (verified in California’s Immunization Registry). The index patient became symptomatic on May 19 with nasal congestion and fatigue. This teacher reported attending social events during May 13–16 but did not report any known COVID-19 exposures and attributed symptoms to allergies. The teacher continued working during May 17–21, subsequently experiencing cough, subjective fever, and headache. The school required teachers and students to mask while indoors; interviews with parents of infected students suggested that students’ adherence to masking and distancing guidelines in line with CDC recommendations (*3*) was high in class. However, the teacher was reportedly unmasked on occasions when reading aloud in class. On May 23, the teacher notified the school that they received a positive result for a SARS-CoV-2 test performed on May 21 and self-isolated until May 30. The teacher did not receive a second COVID-19 test, but reported fully recovering during isolation.

**FIGURE 1:**
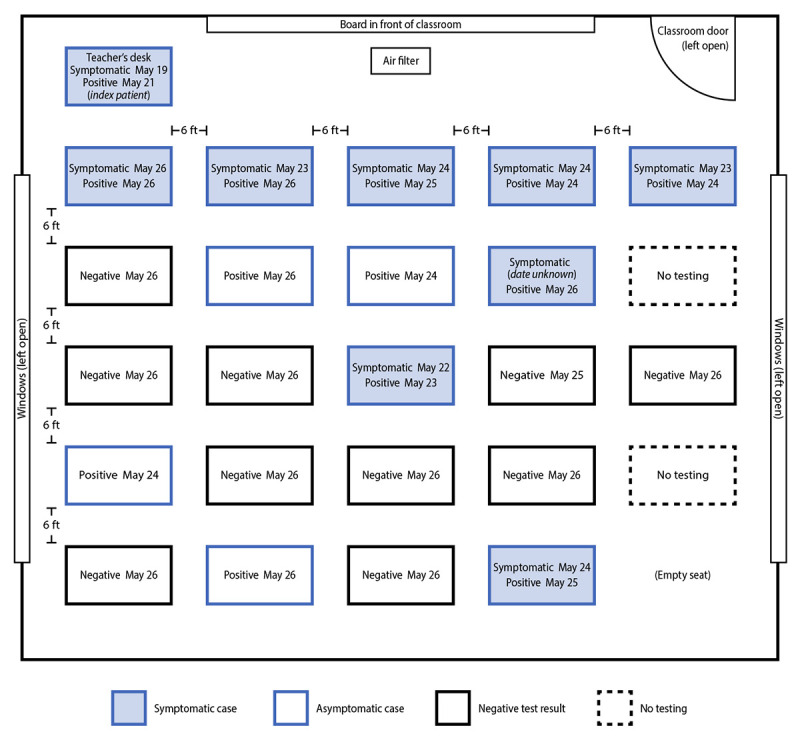
Classroom layout and seating chart for 24 students in index patient’s class, by SARS-CoV-2 testing date, result or status, and symptoms — Marin County, California, May–June 2021

The index patient’s students began experiencing symptoms on May 22. During May 23–26, among 24 students in this grade, 22 were tested. A COVID-19 case was defined as a positive SARS-CoV-2 reverse transcription–polymerase chain reaction (RT-PCR) or antigen test result.* Twelve (55%) of the 22 students received a positive test result, including eight who experienced symptom onset during May 22–26. Throughout this period, all desks were separated by 6 ft. Students were seated in five rows; the attack rate in the two rows seated closest to the teacher’s desk was 80% (eight of 10) and was 28% (four of 14) in the three back rows (Fisher’s exact test; p = 0.036) (Figure 1).

On May 22, students in a another classroom, who differed in age by 3 years from the students in the class with the index case and who were also ineligible for vaccination began to experience symptoms. The two classrooms were separated by a large outdoor courtyard with lunch tables that were blocked off from use with yellow tape. All classrooms had portable high-efficiency particulate air filters and doors and windows were left open. Fourteen of 18 students in this separate grade received testing; six tests had positive results. Investigation revealed that one student in this grade hosted a sleepover on May 21 with two classmates from the same grade. All three of these students experienced symptoms after the sleepover and received positive SARS-CoV-2 test results. Among infected students in this class, test dates ranged from May 24 to June 1; symptom onset occurred during May 22–31.

**FIGURE 2:**
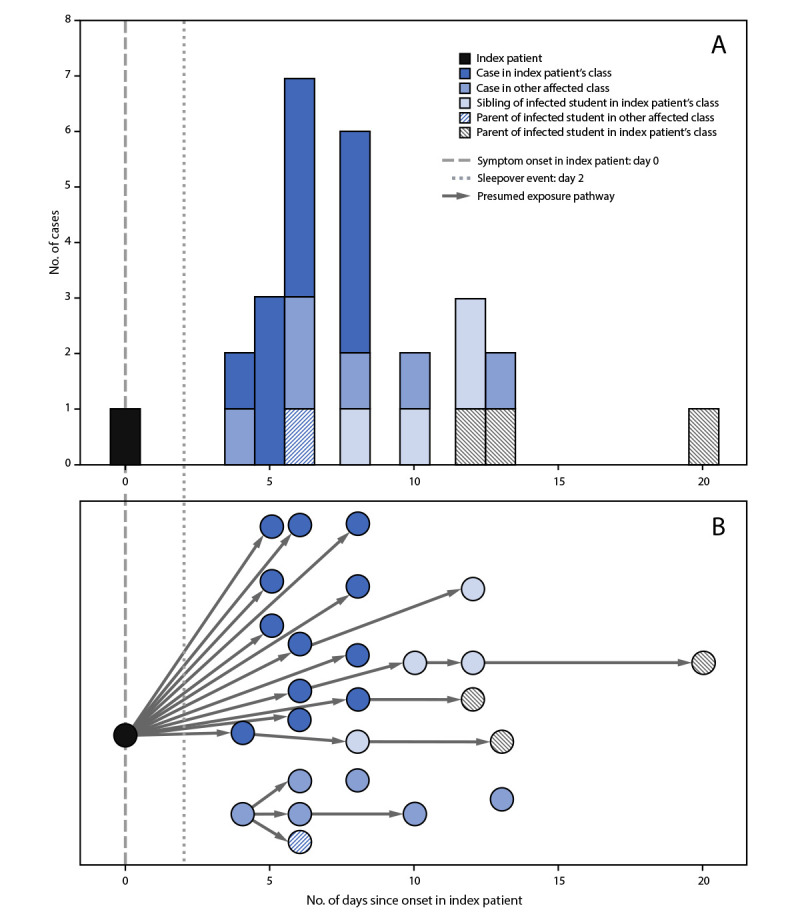
Timeline of SARS-CoV-2 illness onset* after onset in the index patient (A) and presumed transmission**^†^** pathway (B) among students, siblings, and parents, relative to onset in the index patient — Marin County, California, May 2021 * Symptom onset date or specimen collection date, if asymptomatic. ^†^ Presumed transmission based on phylogenetic and epidemiologic analyses.

In addition to the documented infections in the two initial grades, cases were identified in one student each from four other grades. Three patients were symptomatic; dates for testing were May 30 or June 2. These four students were siblings of three students with cases in the index patient’s class, and exposure was assumed to have occurred in their respective homes. In addition to the teacher and 22 infected students, four parents of students with cases were also infected, for a total of 27 cases (23 confirmed by RT-PCR and four by antigen testing) (Figure 2). Among the five infected adults, one parent and the teacher were unvaccinated; the others were fully vaccinated. The vaccinated adults and one unvaccinated adult were symptomatic with fever, chills, cough, headache, and loss of smell. No other school staff members reported becoming ill. No persons infected in this outbreak were hospitalized. This activity was reviewed by Marin County and was conducted consistent with applicable law.

## Public Health Response

**FIGURE 3:**
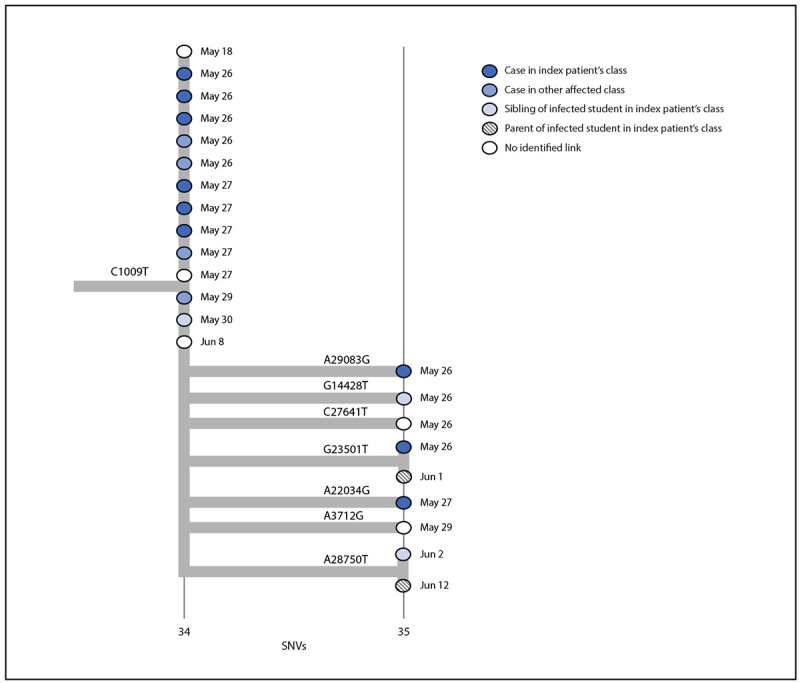
Phylogenetic tree*^,^^†^ of SARS-CoV-2 whole genome sequences and specimen collection dates**^§^** from a COVID-19 outbreak in an elementary school**^¶^** — Marin County, California, May–June 2021 **Abbreviations:** SNV = single nucleotide variant; WGS: whole genome sequencing. * Phylogenetic tree was created with UShER, which uses the Fitch–Sankoff algorithm (a maximum parsimony-based phylogenetic placement approach). https://www.nature.com/articles/s41588-021-00862-7 ^†^ Specimen for the index patient was not available for WGS and is not included on the phylogenetic tree. ^§^ Dates in this diagram reflect the collection date for specimens that underwent WGS; thus dates might differ from those reported in the text for persons whose initial specimens were discarded. ^¶^ Branches are labeled with SNVs; cases (circles) are color-coded to indicate social relationship within the outbreak and labeled with the collection date for the specimen that was sequenced. Vertical lines represent genetically identical viruses; horizontal lines represent genetic descendants with additional SNVs. All sequenced specimens are classified as the SARS-CoV-2 B.1.617.2 (Delta) variant.

On May 26 and June 2, MCPH held testing events at the school as part of outbreak control. During these 2 days, 231 persons were tested, including 194 of 205 students, 21 of 24 staff members and teachers, and 16 parents and siblings of students. The California Department of Public Health assisted with guidance, application of additional prevention strategies, and on-site testing. Community contacts and all students and staff members were encouraged to participate. Specimens for WGS were collected during May 26–June 12; all 18 positive specimens with detectable virus (cycle threshold value <32) were sequenced using ClearDx instruments (Clear Laboratories), Oxford Nanopore MinION sequencing technology, and SARS-CoV-2 ARTIC V3 protocol for amplicon sequencing.^†^ Consensus genome assembly was performed in Terra using Titan Clear Laboratories workflow.^§^ All sequences generated were classified as the Delta variant. A phylogenetic tree was constructed using the UShER pipeline and visualized using Auspice.us^¶^ (*4*) (Figure 3). Eleven sequences were genetically indistinguishable from one another; seven sequences contained additional single nucleotide variations. Among the indistinguishable specimens, six were from students of the index patient, four were from students in the separate grade, and one was from a sibling of a student in the index patient’s class, suggesting that infections occurring in the two grades likely were part of the same outbreak. The epidemiologic link between the two grades remains unknown but is thought to be interaction at the school. Five additional related sequences from community cases (in two adults and three children) were later identified, including three more genetically indistinguishable sequences. One was from an adult with specimen collection 1 day before symptom onset in the index patient. Case investigation records did not establish an epidemiologic link between these five community cases and the school outbreak.

Following the outbreak, infected persons were isolated for 10 days after onset of symptoms (or positive test date for asymptomatic cases). All students with known exposure to an infected person quarantined at home for 10 days following their last known contact. Unvaccinated household and community contacts were directed to quarantine for 10 days following their last known exposure to an infected person, with the option to leave quarantine after 7 days if they remained asymptomatic and received a negative test result from a specimen collected on day 5 of quarantine or later. The two affected classrooms were closed and sanitized during May 21–30 and May 24–June 2, respectively.

## Discussion

This outbreak of COVID-19 that originated with an unvaccinated teacher highlights the importance of vaccinating school staff members who are in close indoor contact with children ineligible for vaccination as schools reopen. The outbreak’s attack rate highlights the Delta variant’s increased transmissibility**and potential for rapid spread, especially in unvaccinated populations such as schoolchildren too young for vaccination. However, transmission to community contacts appeared lower than that of some previously reported Delta variant outbreaks (*5*). Further transmission might have been prevented by high levels of community vaccination; at the time of this outbreak, approximately 72% of eligible persons in the city where the school is located were fully vaccinated.^††^ These findings support evidence that the current COVID-19 vaccines with Food and Drug Administration approval or Emergency Use Authorization are effective against the Delta variant; however, transmission risk remains elevated among unvaccinated persons in schools. In addition to vaccination of eligible persons, implementation of and strict adherence to multipronged nonpharmaceutical prevention strategies including proper masking, routine testing, ventilation, and staying home while symptomatic are important to ensure safe school instruction.

The findings in this study are subject to at least three limitations. First, the teacher’s specimen was unavailable for WGS, which prevented phylogenetic identification of the outbreak’s index patient. Second, testing for parents and siblings was self-directed and took place mostly outside the school setting, which could have led to underascertainment of cases. Finally, challenges in testing acceptance among possible contacts from outside the school led to difficulty in characterizing the outbreak’s actual spread into the community, as is evidenced by later discovery of additional community cases with sequences indistinguishable from those in the school outbreak.

Ineligibility because of age and lack of vaccination contribute to persistent elevated risk for outbreaks in schools, especially as new SARS-CoV-2 variants emerge. However, implementation of multiple prevention strategies within schools can mitigate this risk. The rapid transmission and vaccine breakthrough infections in this outbreak might have resulted from the schoolchildren’s vulnerability because of ineligibility for vaccination, coupled with the high transmissibility of the Delta variant. New evidence of the Delta variant’s high transmissibility, even among fully vaccinated persons (*6*,*7*), supports recommendations for universal masking in schools^§§^ (*1*). Further application of nonpharmaceutical prevention strategies, including routine testing, ventilation, and staying home while symptomatic, are also important for protecting the health of schoolchildren ineligible for vaccination because of their age (*3*). In addition, phylogenetic analysis can help to clarify transmission patterns and characterize outbreak progression. Capacity-building efforts offered by regional and state laboratories enabled more sophisticated analysis at the local level; such efforts might be useful as vaccination rates increase, new variants emerge, and outbreaks become more localized.

**Summary**

**What is already known about this topic?**

The SARS-CoV-2 B.1.617.2 (Delta) variant is highly transmissible. Prevention guidance in schools varies by jurisdiction.

**What is added by this report?**

During May 23–June 12, 2021, 26 laboratory-confirmed COVID-19 cases occurred among Marin County, California, elementary school students and their contacts following exposure to an unvaccinated infected teacher. The attack rate in one affected classroom was 50%; risk correlated with seating proximity to the teacher.

**What are the implications for public health practice?**

Vaccines are effective against the Delta variant, but transmission risk remains elevated among unvaccinated persons in schools. In addition to vaccination, strict adherence to multiple nonpharmaceutical prevention strategies, including masking, are important to ensure safe school instruction.

## Footnotes

*
https://cdn.ymaws.com/www.cste.org/resource/resmgr/ps/positionstatement2020/Interim-20-ID-02_COVID-19.pdf

†
https://www.protocols.io/view/ncov-2019-sequencing-protocol-bbmuik6w

§
https://public-health-viral-genomics-theiagen.readthedocs.io/en/latest/titan_workflows.html

¶
https://auspice.us

** https://www.cdc.gov/coronavirus/2019-ncov/variants/variant-info.html (accessed August 10, 2021)

†† https://coronavirus.marinhhs.org/ (accessed August 10, 2021)

§§ https://www.cdph.ca.gov/Programs/CID/DCDC/Pages/COVID-19/K-12-Guidance-2021-22-School-Year.aspx (accessed August 2, 2021)
